# Study the Effect of the Risk Factors in the Estimation of the Breast Cancer Risk Score Using Machine Learning

**DOI:** 10.31557/APJCP.2021.22.11.3543

**Published:** 2021-11

**Authors:** Sam Khozama, Ali Mahmoud Mayya

**Affiliations:** 1 *Department of Information Technology and Bionics, Pázmány Péter Catholic University, Budapest, Hungary. *; 2 *Department of Computer Engineering, Tishreen University, Lattakia, Syria. *

**Keywords:** Breast cancer, cancer prediction, machine learning, risk factors

## Abstract

**Objective:**

Early prediction of breast cancer is one of the most essential fields of medicine. Many studies have introduced prediction approaches to facilitate the early prediction and estimate the future occurrence based on mammography periodic tests. In the current research, we introduce a novel machine learning tool for the early prediction of breast cancer.

**Methods:**

Three basic resources are used to identify the most essential risk factors; including the BCSC (Breast Cancer Surveillance Consortium) dataset, a medical questionnaire, and multiple international breast cancer reports. The BCSC dataset has been normalized and balanced; consequently, the questionnaire and the medical reports are analyzed in order to define the degree of importance and a potential weight factor of each risk factor. These weights are used to scale risk factors and then the optimizable tree-based ML model is trained using the balanced weighted risk factors datasets.

**Results:**

Three balanced versions of the BCSC dataset are used; oversampled, down-sampled and mixed datasets. Each risk factor has a weight (1, 2 or 4) assigned based on a mathematical modelling of the questionnaire and the international breast cancer reports. The experiments are applied on the weighted and non-weighted versions of the database, and they indicate that the performance increases significantly by using the weighted version of the risk factors. The tests prove that the down-weighting of the non-essential risk factor increases the accuracy and reduces errors. The overall accuracy of the weighted balanced datasets reaches 100%, 95.8% and 95.9% for down-sampled, oversampled and mixed datasets respectively.

**Conclusion:**

Weighting the risk factors of the BCSC dataset improves the performance by increasing the accuracy and reducing the false rejection and false discovery rates for all versions of balanced datasets. The weighting approach can also be used to improve the estimation score of breast cancer by scaling the individual scores of risk factors.

## Introduction

Nowadays, data analysis is one of the most developing fields of computer science due to the fact that the size of datasets is exponentially increasing day after day. Cancer prediction is one of those fields, using data analysis and Machine Learning (ML) algorithms for the estimation of cancer (Faith, 2020) (Patil, 2020; Kamal, 2020). ML techniques can improve the performance of cancer prediction, the estimation accuracy of which has increased significantly (15%-20%) due to using the ML algorithm during the last years (Kourou et al., 2015). Breast cancer prediction itself can be used to define those potentially high-risk women and guide them to improve their lifestyle, avoiding future therapy and costs (Colditz and Wei, 2015).

Cancer prediction is generally based on risk factors (Ahmad and Mayya, 2020). Half of the cancer cases are caused by some known risk factors (Laky, 2020). For breast cancer, many risk factors, such as early menarche, late menopause, obesity, age at first birth, and hormone therapy affect the exposure period of breast tissue to hormones that lead to cancer (American Cancer Society, 2019).

In the field of breast cancer predictions, some studies used the logistic regression approaches (Bernal et al., 2017; Oyewola et al., 2017; Westerdijk, 2018; Teja et al., 2020), while other studies used neural networks (Wang and Yoon, 2015; Kourou et al., 2015; Hou et al. 2020). Other data mining algorithms were used like decision trees (Rajendran et al., 2020), Naïve Bayes methods (Rajendran et al., 2020; Shieh et al., 2016; Williams et al., 2016), Support Vector Machines (Westerdijk, 2018; Mochen and Sundararajan, 2018; Vard et al., 2018), Random Forests (RF) (Oyewola et al., 2017; Westerdijk, 2018; Hou et al., 2020; Rajendran et al., 2020), optimization algorithms (Vard et al., 2018), etc.

For breast cancer estimation research, many datasets, like the Breast Cancer Surveillance Consortium dataset (BCSC dataset, 2021), consisting of 280,660 records, had been used in many pieces of research (Rajendran et al., 2020; Shieh et al., 2016; BCSC dataset, 2021; Williams et al., 2016). Another international dataset is the Breast Cancer Information Management System (BCIMS) dataset, consisting of 16,000, cases (Peng et al., 2016) and was used by many studies such as Hou et al. (2020) and Zhong et al. (2020). Some other researchers collected their datasets from specialized medical centers or hospitals (Ming et al., 2020; Barlow et al., 2006).

Shieh et al., (2016) proposed a breast cancer prediction model using the information of the clinical and polygenic risks. The Bayes estimation and conditional logistic regression models are used together to study the common effect of ordinary and polygenic risk factors on the future risk of breast cancer. The researchers used 486 cases of the BCSC dataset and found that the prediction accuracy increased from AUC=0.62 to AUC=0.65 after adding the polygenic risk to the model. They concluded that 18% of the cases were classified as high-risk cases in the common model, while it was only 7% for the ordinary risk factors model.

Li and Sundararajan (2018) applied several ML approaches for the prediction of breast cancer. They used only 10,000 cases and eight risk factors of the BCSC dataset. SVM and Bayes classifiers were used for the final risk estimation and got accuracy results of 96.6% and 91.26% for SVM and Bayes classification, respectively.

In 2020, Rajendran et al., (2020) used the supervised ML algorithms on imbalanced class data for the prediction of breast cancer on the BCSC dataset. In order to perform balancing, they used three approaches: Synthetic Minority Oversampling, under-sampling and fusion of both techniques. They also used Bayes classifier, Bayes networks, Random Forests (RF), and random trees as classifiers. The best accuracy they obtained was 99.1%, under False Positive (FP) equals 21%. The problem with this research was that they used only 10,252 instances after applying the balancing techniques; besides that, the results showed low sensitivity of 78.1%.

A new model for predicting breast cancer in Chinese women had been introduced by Hou et al., (2020). They used 7,127 cases of the Breast Cancer Information Management System (BCIMS) dataset and chose specific risk factors based on the fact that they must be known and collected by the same measurement techniques. Consequently, 10 risk factors had been chosen and different prediction models were used, like RF, deep neural networks DNN and XGBoost. They got an accuracy of 72.8 for both DNN and RF, while the XGBoost accuracy was 74.2%.

The Evaluation of many ML classifiers for the prediction of breast cancer under incomplete datasets was suggested by Teja et al., (2020). They evaluated the RF, Logistic Regression (LR) and custom Neural Network (NN). The Area Under Curve (AUC) was used for the performance evaluation on the BCSC dataset. AUC achieved 0.645, 0.634 and 0.649 for LR, RF and NN respectively.

Ming et al., (2020) collected a breast cancer prediction dataset from Geneva University Hospitals. Their dataset included 112587 individuals and 14 variables. They applied different ML algorithms (like the Markov mixed model, adaptive boosting and RF) and obtained accuracy between 84.3% and 88.9%. However, the dataset variables related not only to breast cancer but also to other tissues, so that more risk factors needed to be included.

Most previous studies did not consider the nature of the used breast cancer dataset. Each dataset has some properties that must be understood in order to get a proper accurate estimation as mentioned by the BCSC dataset (2021). For example, the BCSC dataset needs to consider the “count” as a very important variable in order to achieve correct results. Besides that, the BCSC dataset is unbalanced, so it needs a balancing step before any estimation model. The research aim is to develop a new tool for predicting breast cancer based on BCSC risk factors. We have taken into account the “count” variable for good estimation. In addition to that, balancing has been applied as a pre-processing step. The last new option that have been done is the weighting mechanism, in which a weight number of each risk factor is assigned in order to enhance the performance. The following paragraphs will contain the used materials and the proposed approach in detail. Finally, the results and discussion section will be introduced.

## Materials and Methods

*Dataset*

In the current research, the BCSC dataset is used. It includes 280و660 records and 12 risk factors, which are described in Table 1. Besides these risk factors, the dataset includes a variable called “count”, which holds the frequency of each record within the dataset, as mentioned in the BCSC dataset (2021).

*Proposed system*

**Figure 1:**
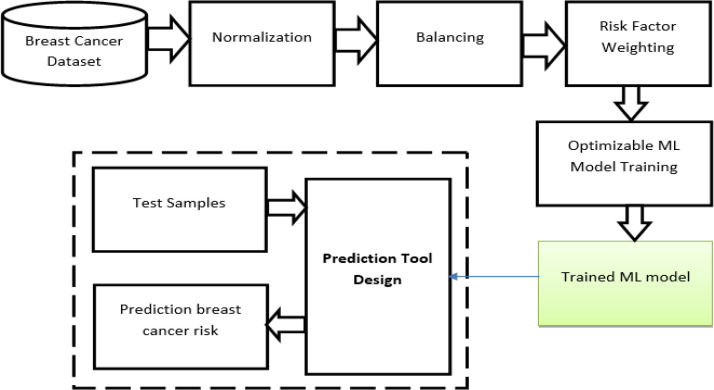
Proposed System Methodology

The proposed risk-estimation model of breast cancer is described in Figure 1 so that the BCSC dataset is obtained from http://www.bcsc-research.org/, and all risk factors are used. First, the dataset is normalized to ensure that all risk factors initially have the same effect on the final risk estimation. The normalization is done using Equation 1:

Risk_factor_i_=Risk_factor_i_/max(Risk_factor_i_)                               (1)

i=1,2,…,M 

Where M is the number of risk factors. The normalization step makes the value of each risk factor ranging from 0 to 1.

The second step is balancing, in which the dataset must be manipulated in order to achieve the balancing between target categories. The original BCSC dataset has two target categories (0: no cancer, 1: cancer). While the “1” category has only 3.32%, the “0” category has 96.68% of all samples, which is why the BCSC is extremely unbalanced. The minimal appearance of the minor category in the unbalanced datasets leads any classifier to generate inaccurate predictions due to the inappropriate training (Somasundaram and Reddy, 2016). So for the original BCSC dataset, any classifier can produce more than 96% accuracy only if it has recognized all “0” class samples, even if all “1” class samples have been incorrectly estimated as “0”. To solve this problem, the so-called balancing mechanism is applied. Three different balancing approaches were applied. The first is oversampling, in which the samples of the minor class are duplicated many times so that their percentage increases. Duplication will enhance the training significantly. The second approach is down-sampling, in which some of the majority-class samples are removed until decreasing its percentage to the required value. As for the last approach, duplicating the minor class samples and eliminating some of the majority class samples are performed until achieving the desired balance.

To perform the third step, the weighting algorithm is applied. To achieve a good, accurate weighting, two branches were taken. First, a questionnaire of the risk factors listed in the BCSC dataset was created. The aim of this questionnaire was to establish medical knowledge, so it was sent to 40 specialist physicians working in the field of cancer treatment and diagnosis. Afterward, the results of the questionnaire were analyzed to define the expert’s opinion of the impact of each risk factor in the final score of cancer risk. The second branch of this study investigated the international medical reports from which the recent discussions of breast cancer risk factors were obtained to define the impact from another point of view. 

*Machine Learning Model selection*

After getting the final impact (weight) of each risk factor, the final step is the selection of the ML model. Multiple ML prediction algorithms are available, but the optimization tree model is chosen due to its ability to tune the hyperparameters, deal with missing or noisy data, and handle redundant attributes values (Apté and Weiss, 1997; Mantovani et al., 2018). The decision tree algorithm first considers all samples of the dataset as the root node. The basic challenges are selecting the best attribute to be the root node and then deciding to split the node into all attributes and select the one with the best split performance. Decision trees actually compute the Information Gain (IG) as illustrated in Equation 2 (Kelleher, 2020) across all possible attributes and then choose the attribute with the lowest IG. This means that the selected attribute is the one that separates the training samples the best.

$\mathrm{IG}\left(\mathrm{T. a}\right)=H(\mathrm{T0}-H\left(T|a\right)=-\sum_{i=1}^{k}p_{i}\log_{2}\left(p_{i}\right)-\sum_{i=1}^{k}p_{r}(i|a)\log_{2}(p_{r}\left(i|a\right))$              (2)

Where H(T) is the entropy of the parent node of the tree T, H(T|a) is the entropy of the child node a (attribute a), k is the number of subsets generated by each split, pi is the percentage (probability) of class i in the node T, pr(i|a) is the percentage of class i given that the split child (attribute) is a.

Now, for the optimizable tree classifier, three different parameters are tuned. These parameters are the criterion (the attribute selection measure), the splitter (the split strategy) and the maximum depth of a tree.

*Prediction Tool Design*

**Figure 2:**
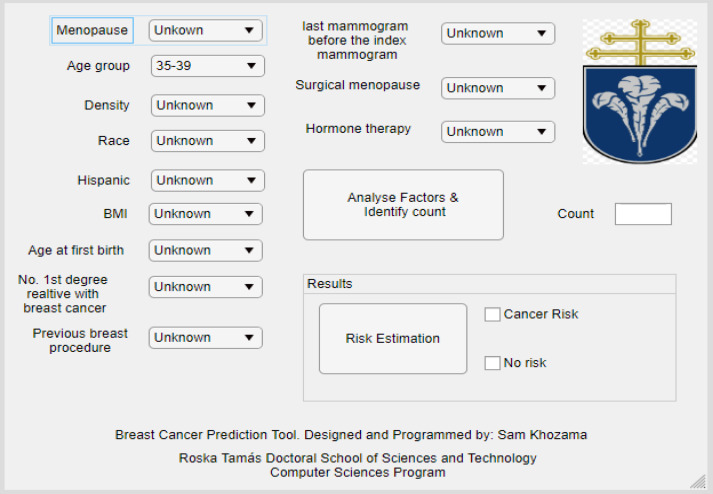
Proposed Breast Cancer Prediction Tool

After getting the final optimizable decision tree classifier, the tool is built based on the trained model. The tool is designed using MATLAB App designer. Figure 2 shows the designed tool. After entering the values of all factors except “count”, the tool searches into the BCSC dataset to find the match between the entered risk values and all records of the dataset. If a match is found, the corresponding count is considered as the count of the test sample. Otherwise, the count will be 1.

## Results

*The preprocessing of the risk estimation dataset*

Preprocessing of the risk estimation dataset includes two basic steps, which are normalization and balancing. Table 2 includes the results of the suggested balancing methods, where the majority class label is 0(no cancer), and the minor class label is 1 (cancer risk). 

For the oversampling approach, the “1” minor class has been duplicated five times until its percentage became 14.64%, while the majority-class percentage became 85.36%. For the down-sampling approach, we minimized the majority-class samples by a factor (3.524 times) until getting a 10.78% percentage for the minor class and 89.22% for the majority one. For the last approach, we duplicated the minor class samples and eliminated some of the majority class samples until getting 17.1% and 82.9% for the minor and majority classes, respectively.

*Risk factor weighting results*

In order to obtain the risk factors weights, the results of the questionnaire are analyzed and the questionnaire-based degree of importance (DOI^q^_i_) of each risk factor is defined based on Equation 3:

${\mathrm{DOI}}_{i}^{q}=H_{i}\mathrm{*0}∙6+M_{i}\mathrm{*0}∙4$ (3)

Where H_i_ and M_i_ are the high-risk and medium-risk percentages of the risk factor i shown in Table 3.

Based on the analysis of the questionnaire, the following results are inferred:

The risk factors with the largest high-risk levels are number of first degree relatives with breast cancer (nrelbc), hormone therapy.

Age, menopause, density and race are the risk factors with the largest medium-risk levels.

Hispanic, breast procedure (brstproc), and surgical menopause have the lowest risk levels.

Factors with a high DOI (more than 0.4) are nrelbc, age, hormone therapy.

Some other risk factors like age at first birth, menopause, density, Body Mass Index (BMI), last mammogram before the index mammogram (lastmamm) and race have medium DOI (between 0.3 and 0.4) are

Other risk factors with a low DOI (less than 0.3) are Hispanic, brstproc and surgical menopause.

The international medical reports, on the other hand, indicate other opinions. So, we concluded the information about risk factors, and then this information was compiled and classified according to the number of times the factors were mentioned in the list of the essential risk factors (Ess_Numi), then within the list of the secondary-risk factors (Sec_Numi), and the risk degree DOI^R^_i_ was calculated according to Equation 4:



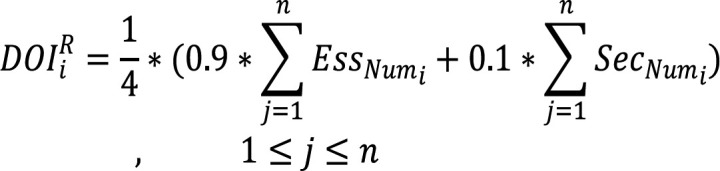



(4)

Where n is the number of medical studies that have been analyzed and the denominator (4) is the maximum DOI. Based on the analysis of the previous studies and previous breast cancer medical reports, like Breast Cancer Facts & Figures (2019), Cancer Facts & Figures (2020), Breast cancer risk factors (2009) and Breast Cancer Risk and Prevention (2019), we supposed that 90% of the essential risk factor effect and 10% percent of the secondary risk factors will be summed to constitute the final DOIR value.

The final DOI (DOI^F^_i_) is inferred from the medical questionnaire-based degree of importance (DOI^Q^_i _of Table 3) and the international medical reports-based degree of importance (DOI^R^_i_of Table 4) as Equation 5 suggests, while the suggested training weight (STW) in Table 5 is inferred based on the DOIF (Equation 6).



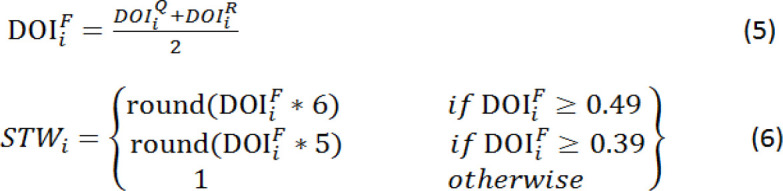



The most significant risk factors, as Table 5 describes, are Age group, nrelbc and race while the medium significance risk factors are: Hormone therapy, agefirst, density, Menopause and BM. However, the least essential risk factors are Hispanic, brstproc, lastmamm and surgical menopause.

The effect of weighting the risk factors against the non-weighted version of the dataset is shown in Table 6. The results indicate that the performance increases by 6.9% after the weighting approach; similarly, the False Discovery Rate (FDR) is minimized by 22.6% and 3.2% for the minor and majority class respectively. The False Negative Rate (FNR), as well, is minimized for the majority and minor class by 5% and 17.6% respectively. FDR and FNR indicate the percentage of false positives and false negatives respectively (Pawitan et al., 2005).

**Table1:** Dataset Risk factors Description

No.	Risk Factor	Description
1	Menopause	Pre=0(23.47%), Post or age>55=1(68.65%), Unknown=9(7.6%)
2	Age group	Group1=35-39(1.79%); Group2=40-44(12.1%); Group3=45-49(16.18%); Group4=50-54(17.9%); Group5=55-59(13.96); Group6 =60-64(11.1%); Group7=65-69(9.69%); Group8=70-74(8.49%); Group9=75-79(6.06%); Group10=80-84(2.91%).
3	Density	Breast density: Almost entirely fatty: 1(6.19%), Scattered fibro-glandular densities:2(32.69%), Heterogeneously dense:3(28.17%), Extremely dense:4(5.68%), 9:Unknown or other indexes(27.26%)
4	Race	1=white(72.63%); 2=Asian/Pacific Islander(4.3%); 3=black(5.08%); 4=Native American (1.19%); 5=other/mixed(0.9%); 9=unknown(15.87%)
5	Hispanic	No:0(73.1%) Yes:1(6.58%), Unknown:9(20.3%)
6	BMI	1=10-24.99(21.27%); 2=25-29.99(13.6%); 3=30-34.99(6.05%); 4=35 or more(3.25%); 9=unknown(55.83%)
7	Age at first birth (agefirst)	0=Age<30(30.18%); 1=Age 30 or greater(5.9%); 2=Nulliparous(8.41%); 9=unknown(55.51%)
8	Number of first degree relatives with breast cancer (nrelbc)	0=zero(71.81%); 1=one(12.36%); 2=2 or more(0.65%); 9=unknown(15.18%)
9	Previous breast procedure (brstproc)	0=no(71.97%); 1=yes(17.57%); 9=unknown(10.46%)
10	last mammogram before the index mammogram (lastmamm)	0=negative(75.22%); 1=false positive(1.42%); 9=unknown(23.36%)
11	Surgical menopause	0=natural(30%); 1=surgical(17.86%); 9=unknown or not menopausal(52.14%)
12	Hormone therapy	0=no(30.47%); 1= yes(28.56%); 9=unknown (40.97%)
13	Count	Frequency of each record in the dataset

**Table 2:** Balancing Approaches and Their Corresponding Classes Percentages

Balancing method	Majority class sample number	Majority class percentage	Minor class sample number	Minor class percentage
Oversampling	271,355	85.36%	46,525	14.64%
Down-sampling	77,000	89.22%	9,305	10.78%
Mixed	225,562	82.90%	46,525	17.10%

**Table 3.** Breast Cancer Risk Factors DOI Based on the Medical Questionnaire

No.	Risk Factor	High	Median	Low	DOIQ
1	Menopause	30%	47.50%	22.50%	0.37
2	Age group	27.50%	62.50%	10.00%	0.415
3	Density	25%	45.00%	30.00%	0.33
4	Race	25%	40.00%	35.00%	0.31
5	Hispanic	19.40%	16.70%	63.90%	0.183
6	BMI	25.60%	38.50%	35.90%	0.307
7	agefirst	27.50%	45.00%	27.50%	0.345
8	nrelbc	56.40%	25.60%	17.90%	0.44
9	brstproc	34.20%	23.70%	42.10%	0.30
10	lastmamm	34.20%	32.10%	33.70%	0.33
11	Surgical menopause	7.70%	30.80%	61.50%	0.169
12	Hormone therapy	42.50%	37.50%	20%	0.405

**Table 4:** Breast Cancer Risk Factors DOI Based on the Medical Reports

No.	Risk Factor	Essential†				Secondary				DOIR
		1	2	3	4	1	2	3	4	
1	Menopause			1		1	1	1		0.3
2	Age group	1	1	1	1					0.9
3	Density	1	1					1	1	0.5
4	Race	1	1		1					0.675
5	Hispanic	1							1	0.25
6	BMI		1			1		1	1	0.3
7	agefirst		1	1		1			1	0.5
8	nrelbc		1	1	1	1				0.7
9	brstproc					1		1		0.05
10	Lastmamm									-
11	Surgical menopause					1				0.025
12	Hormone therapy	1	1					1	1	0.5

**Table 5:** The DOIf of the Breast Cancer Risk Factors

No.	Risk Factor	DOIf †	STW
1	Menopause	0.3357	1
2	Age group	0.65751	4
3	Density	0.4156	1
4	Race	0.49253	3
5	Hispanic	0.21659	1
6	BMI	0.30358	1
7	agefirst	0.42255	2
8	nrelbc	0.572	3
9	brstproc	0.1751	1
10	lastmamm	0.16511	1
11	Surgical menopause	0.09712	1
12	Hormone therapy	0.45254	3

†Numbers 1-12 indicates the weight order.

**Figure 3:**
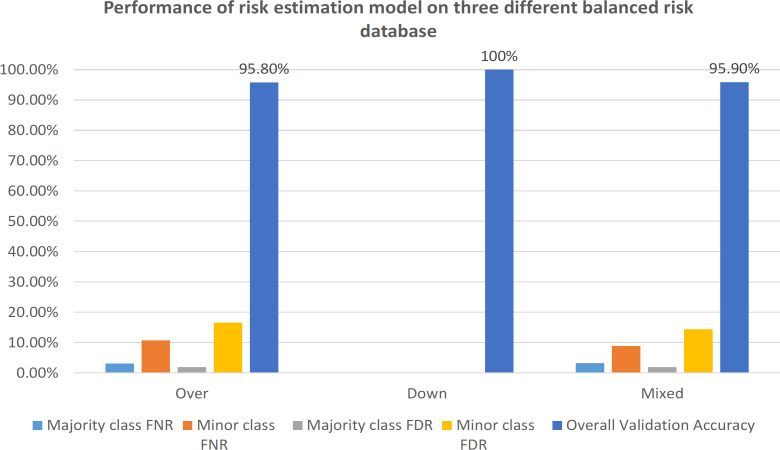
Effect of Scaling Risk Factors on the Performance of Risk Estimation Model on Three Different Balanced Database

**Table 6:** Evaluation of the Risk Estimation Model Using the Weighted and Non-Weighted Version of the Risk Factors

Data status	Majority class FNR	Minor class FNR	Majority class FDR	Minor class FDR	Overall Validation Accuracy	Training Time
With Weighting	3.30%	10.50%	1.80%	17.50%	95.70%	38.65
Without Weighting	8.30%	28.10%	5.00%	40.10%	88.80%	41.09

**Table 7:** Evaluation of the Risk Estimation Model Using Different Selections of the Weighted Risk Factor

Deleted risk factor	Majority class FNR	Minor class FNR	Majority class FDR	Minor class FDR	Overall Validation Accuracy	Training Time
Age	3.50%	16.40%	2.80%	19.60%	94.60%	36.05
Race	6.40%	21.60%	3.80%	32.10%	91.40%	43.23
Nrelbc	4.90%	16.10%	2.80%	25.40%	93.50%	39.09
Hormone Therapy	4.00%	13.10%	2.30%	21.20%	94.70%	38.10
surgmeno	3.90%	13.10%	2.30%	20.80%	94.70%	38.20
lastmamo	4.70%	15.30%	2.70%	24.40%	93.80%	40.32
brstproc	4.30%	13.70%	2.40%	22.60%	94.30%	37.12
agefirst	4.80%	17.40%	3.00%	25.50%	93.30%	41.37
bmi	4.90%	16.60%	2.90%	25.70%	93.40%	39.70
Hispanic	4.90%	16.50%	2.90%	25.60%	93.40%	41.37
Density	4.40%	15.10%	2.60%	23.00%	94.10%	40.20
menopause	3.50%	11.30%	2.00%	18.80%	95.30%	39.20
Age & Race	6.60%	33.50%	5.80%	36.60%	89.50%	36.07
Race & Nrelbc	7.80%	30.50%	5.40%	39.50%	88.90%	36.20
Nrelbc & Age & Race	5.50%	50.50%	8.40%	39.40%	87.90%	33.59
menopaus & brstproc & surgmeno	5.40%	20.90%	3.70%	28.50%	92.30%	33.70

**Table 8:** The Effect of Down-Weighting the Weak-Impact Risk Factors on the Performance of Risk Estimation Model on the Oversampled Risk Database

Down-scaling	Majority class FNR	Minor class FNR	Majority class FDR	Minor class FDR	Overall Validation Accuracy
menopause=0.5	3.10%	10.80%	1.90%	16.60%	95.80%
menopause=0.5,brstproc=0.2	3.10%	10.70%	1.90%	17.00%	95.80%
menopause=0.5,brstproc=0.2, ,lastmamm=0.2	3.20%	11.50%	2%	17.50%	95.60%
brstproc=0.2,lastmamm=0.3, surgmeno=0.2	3.30%	10.10%	1.80%	17.60%	95.70%
menopause=0.5,brstproc=0.2, lastmamm=0.3,surgmeno=0.2	3.10%	11%	1.90%	17%	95.70%
menopause=0.5,Density=0.3, brstproc=0.2,lastmamm=0.3, surgmeno=0.2	3.10%	10.70%	1.90%	16.60%	95.80%

## Discussion

To check the results shown in Table 6, many test scenarios are suggested by removing one or more essential/non-essential risk factors; so that the optimizable tree-based classifier accuracy, as well as the classification errors, are computed to check the validity of each scenario.

Table 7 illustrates that the weighted version of the dataset has better performance than the non-weighted one. Weighting the risk factors has increased the performance by 6.9%. The risk factors differ in their degree of importance (i.e., their effect in defining the final risk degree). Table 7 shows that the most effective risk factor is the “Race” factor as the accuracy decreased by 4.3% after removing this factor. Other risk factors like age at first birth (agefirst), age group, Nrelbc, BMI and Hispanic affect the performance significantly after removing them from the dataset. By removing one of the risk factors (race, age group, agefirst, BMI and Hispanic), an increment in the minor FNR rate is noticed. By removing couples of risk factors like (age and race) or (Nrelbc, age and race) the performance degrades significantly by 6.2 to 7.8% and the minor class FNR error increases as well by 23% to 40%, which is a very huge error rate (i.e. these factors are essential). However, some factors like menopause, surgical menopause (surgmeno) and hormone-therapy decrease the accuracy by a small range (0.4% to 1%). From another point of view, missing the three risk factors (menopause, brstproc and surgmeno) decreases the accuracy only by 3.4%. So these factors have less impact than others on defining the last risk degree, and in order to validate this conclusion, a down-weight approach was applied in which each weak-impact risk factor is weighted by a less-than-1 factor (0.2, 0.3, 0.5, etc.) and the results are listed in Table 8. Scaling menopause, for example, by 0.5 improves the validation accuracy by 0.1%. Scaling the other low-important risk factors also improves the accuracy by 0.1% and reduces the FNR error by 0.2%. However, in some cases; it increases the FNR of the minor class (and this is because the minor class percentage is small), but at the same time the FDR rate has been decreased by (0.5-0.9%).

The same scaling technique used on the oversampled dataset; is applied to the down-sampled and the mixed ones, Figure 3 includes a detailed comparison of the performance of scaling choice (age=4, race=3, agefirst=2, nrelbc=3, current hormone therapy (current_hor)=3, menopause=0.5, density=0.3, brstproc=0.2, lastmamm=0.3, surgmeno=0.2) over the three balanced datasets. Figure 3 shows that the down-sampled dataset has the highest accuracy (100%) and the least error rates (0%); however, this down-sampled dataset has a volume of 27.15% only compared with the over-sampled version. So although the down-sampled dataset has the best accuracy, the over-sampled and the mixed versions have better performance since they consist of a much larger number of samples so that the new test samples will be classified more correctly.

In this research, the effect of weighting and selection of the risk factors has been studied. In addition, three versions of the balanced dataset were tested. The experiments proved that the weighting technique improved the accuracy and reduced the errors significantly. In future work, the weighting model will be used to generate a fuzzy risk factor score in the range (0-100) instead of a scalar risk score.

## Author Contribution Statement

Khozama S. proposed the idea and design of the work. Mayya A. collaborated in editing the layout of the paper and coding some parts of the software. Both authors contributed in formulating the mathematical equations, writing the manuscript, and testing the software.

## Funding Statement

This work was supported by Pázmány Péter Catholic University, Budapest, Hungary. The research project has been partially supported by the European Union, co-financed by the European Social Fund through the grant EFOP-3.6.3-VEKOP-16-2017-00002.

## Approval

The current study deals with secondary public data (breast cancer risk factors), so it doesn’t need from approval of scientific body. This paper is a part of the dissertation submitted in fulfillment of the requirements for the degree of Doctor of Philosophy in Pázmány Péter Catholic University, Budapest, Hungary.

## Ethics approval

Dataset for this study is completely public; therefore, no ethical approval is required. 

## Availability of data

Data collection and sharing was supported by the National Cancer Institute-funded Breast Cancer Surveillance Consortium (HHSN261201100031C), available at: http://www.bcsc-research.org/.

The study is not registered in any registering dataset.

## Conflict of interest

The authors declared no conflicts of interest.
